# Endothelial and smooth muscle cell interaction with hydrothermally treated titanium surfaces

**DOI:** 10.1007/s44164-024-00073-4

**Published:** 2024-07-19

**Authors:** Vignesh K. Manivasagam, Ketul C. Popat

**Affiliations:** 1 Department of Mechanical Engineering, Colorado State University, Fort Collins, CO USA; 2 School of Advanced Materials Discovery, Colorado State University, Fort Collins, CO USA; 3 School of Biomedical Engineering, Colorado State University, Fort Collins, CO USA; 4 Department of Bioengineering, George Mason University, Fairfax, VA USA

**Keywords:** Titanium, Hydrothermal treatment, Coronary stents, Surface modification, Surface characterization, Endothelization and atherosclerosis

## Abstract

Cardiovascular diseases (CVDs) remain the leading cause of death worldwide, and the most common form is coronary artery disease (CAD). Treatment options include coronary artery bypass surgery (CABG) or percutaneous heart intervention (PCI), but both have drawbacks. Bare metal stents (BMS) are commonly used to treat CAD; however, they lead to restenosis. Drug-eluting stents (DES) were developed to overcome this limitation; however, they lead to late thrombosis. Hence, there is an urgent need to engineer stent surfaces that selectively prevents smooth muscle cell adhesion and proliferation (restenosis), while promoting endothelial cell adhesion and differentiation (endothelialization), thus enhancing hemocompatibility. In this study, hydrothermal treatment with either sodium hydroxide or sulfuric acid was used to modify the surface of titanium. Titanium surface treated with sulfuric acid led to a micro-nano-surface morphology that selectively promoted endothelial cell adhesion and differentiation while prevented smooth muscle cell proliferation.

## Introduction

Cardiovascular diseases (CVDs) continue to be the leading cause of the death in the United States and worldwide. According to the Global Burden of Disease study, 17.8 million deaths globally are due to CVDs [1]. Coronary artery disease (CAD) is the most common type of CVD, caused by plaque buildup on the walls of arteries. This plaque is mostly made of cholesterol deposits. Arteries play a major role in circulating blood inside our body and this plaque buildup narrow down the blood vessels and limits the blood flow. This disease is also called atherosclerosis and a common treatment for this disease is either coronary artery bypass surgery (CABG) or percutaneous heart intervention (PCI) [2]. During CABG, a vascular graft is attached to the affected artery to restore the blood flow [3]. This is an extensive surgical and invasive procedure; however, this procedure can lead to hemorrhage, infection, and pneumonia [4]. During PCI, a metallic stent is placed on the affected region of the artery to restore the blood flow. This is a nonsurgical and minimally invasive technique; however, this procedure can lead to inflammation and hemorrhage [5, 6]. But the recovery time from CABG is higher than PCI. Hence, PCI is preferred over CABG due to its simplicity [7].

Bare metal stents (BMS) were the first kind of stents used in the 1980s and 1990s [8]. These are mostly made of nickel-titanium, stainless steel, and cobalt-chromium alloy for their excellent strength and corrosion [9]. The major limitation of BMS is restenosis [10]. Restenosis is a gradual re-narrowing of the vessel in the stented area [11]. In-stent restenosis is a complex process involving tissue repair after vessel injury during stent implantation leading to neo-intimal hyperplasia, where the smooth muscle cells proliferate and migrate into the internal layer [12]. These smooth muscle cells interact with the implant surface and de-differentiate and initiate proliferation. This will reduce the lumen diameter obstructing the blood flow [13]. Because BMS had high potential for early-stage restenosis, the development of drug eluding stents (DES) had emerged [14]. DES are commonly used as they have molecular therapy to reduce restenosis, inflammation, and initial thrombus formation [15, 16]. DES are BMS that are coated with polymer overlayer containing specific drugs such as sirolimus, paclitaxel, everolimus, and zotarolimus [17]. However, the side effect of this is impaired endothelial regeneration, depletion of drug with time, and late thrombosis [18, 19]. Thrombosis is acute syndrome where the blood clots in the surface of the stent and, once the clotting cascade begins, it spreads rapidly, and this increase the chances of mortality [20]. Hence, it is vital to develop an implant surface that prevents thrombosis. Regardless of the kind of stents in current market, endothelization is a major limitation. Therefore, there is a crucial need to develop a stent surface that can selectively prevent smooth muscle cell adhesion and proliferation (restenosis), simultaneously promote endothelialization for enhancing blood compatibility.

In a healthy individual, the endothelial lining in blood vessels prevent adhesion of cells and clotting of proteins to maintain hemostasis, a similar strategy can be adopted to develop new implant surfaces that promote endothelial cells adhesion, proliferation, and differentiation [21]. Researchers have taken various approaches to develop hemocompatible surfaces for cardiovascular implants. Studies have demonstrated the influence of various surface properties such as morphology, chemistry, and wettability on cell and blood interaction [22–24]. Hence, researchers have either modified surface morphology or surface chemistry or both for enhancing hemocompatibility and simultaneously improve endothelization. Anodization is one of the surface modification technique that has been extensively researched as it leads to higher hemocompatibility with decreased platelet and smooth muscle cell adhesion as well as enhanced endothelial cell growth through the formation of titania nanotubes [25–27]. Further, studies carried out on titania nanotubes coated with zinc and polymers showed lower smooth muscle cell adhesion and higher endothelial cell adhesion. However, oxidized nanotubes and coatings are prone to delamination and cracks [28, 29]. Researchers have also explored several surface modification approaches for improving endothelization on titanium surface, such as coating with peptides derived from proteins that are extracted from extracellular matrix. Protein active sequences such as c[RGDfK], SIKVAV, and VGVAPG have been coated on titanium surfaces. Results showed enhanced endothelial cell adhesion and differentiation [30]. Similarly, poly (4-methyl-1-pentene) (PMP) gas-exchange membranes coated on titanium surfaces using the pulsed vacuum cathodic arc plasma deposition (PVCAPD) technique promoted a stable endothelial surface without promoting inflammation and the thrombogenicity [31]. Hence, there is a critical demand to develop a stable surface with selective endothelial adhesion and preventing smooth muscle cells.

To address this critical need, in this study, micro/nano-surface morphologies were developed on titanium surface using hydrothermal treatment with sodium hydroxide and sulfuric acid. The modified surfaces were characterized for their morphology using a scanning electron microscope (SEM) and the wettability was evaluated through contact angle measurement with water using a goniometer. Cytocompatibility of the surfaces were assessed by evaluating cytotoxicity, cell viability, adhesion, proliferation, morphology, and differentiation of smooth muscle cells and endothelial cells. Results indicated that hydrothermal treatment with sodium hydroxide and sulfuric acid led to the formation of two unique surface morphologies. Sodium hydroxide surface had a planar surface with fibrous structure enabled both endothelial and smooth muscle cell proliferation and differentiation. However, the 3D polygon structure with nano-pits prevented the bigger smooth muscle cell proliferation and differentiation. Further, surface wettability studies showed that both the surfaces were significantly hydrophilic compared to unmodified titanium surface. Cytotoxicity studies revealed that both modified surfaces did not induce any toxicity to both smooth muscle cells and endothelial cells after incubation for 24 h. Cell studies indicated that the micro-nano-surface morphology developed by sulfuric treatment selectively prevented smooth muscle cell adhesion and promoted endothelial cell adhesion and proliferation, thus may be a potential surface for cardiovascular implants.

## Materials and methods

### Fabrication of nanoporous structures on titanium surfaces

Commercial pure medical grade II titanium sheets were cut into 5 cm × 3 cm × 0.02 cm surfaces. These surfaces were mechanically polished using silicon-carbide dry abrasive sheets. The grit size of the abrasive sheets was gradually increased (400, 600, 800, 1000, 1200) to attain a polished surface. Polished surfaces were sonicated for 10 min in an acetone medium and these surfaces were subsequently rinsed with water and air dried. They were treated as follows:

- Sodium hydroxide treatment: cleaned surfaces were submerged in a sodium hydroxide base solution (5 M) and hydrothermally treated at 80 °C in a hot air oven for 8 h. Experimental parameters such as temperature, solution concentration, and duration of experiment was tweaked to develop different surface morphology and tested for hemocompatibility. The surface with the highest hemocompatibility as explained in the author’s previous work was used in this manuscript [32]. After being treated, the modified substrates were annealed for 1 h at 300 °C.
- Sulfuric acid treatment: cleaned surfaces were submerged in a sulfuric acid solution (0.5 M) and hydrothermally treated at 80 °C in a hot air oven for 8 h. Experimental parameters are optimized for surface morphology as explained in the author’s previous work [33]. After being treated, the modified substrates were annealed for 1 h at 300 °C.

Hydrothermally modified surfaces were further sonicated for 10 min in DI water to remove debris and dried using compressed air. These modified surfaces were stored in a desiccator until further studies. For cell studies, polished and hydrothermal surfaces were further cut down into smaller square surfaces (0.5 cm × 0.5 cm). The following notations were used to refer the treated titanium surfaces: Ti for unmodified titanium surfaces (control), Ti_H2SO4_ for sulfuric acid modification, and Ti_NaOH_ for sodium hydroxide modification.

### Surface characterization

Morphology of control and modified surfaces was visualized using a JEOL 6500 field emission scanning electron microscopy (FE-SEM). The specimen chamber was maintained at a pressure of 10-5 Pa and operated at 15 kV accelerating voltage. Images were taken at different magnification 500×, 2000×, and 10,000×. The advancing contact angle (θ*) of different surfaces with Milli-Q water was calculated using a Ramé-hart 260F4 goniometer at room temperature. The advancing contact angle was measured by placing a Milli-Q water droplet and the volume was increased in a controlled manner using a micro syringe until the contact angle value saturates.

### Cell culture

Human Umbilical Vascular Endothelial Cells (HUVECs, passage 4) were suspended in endothelial cell basal media with supplement kit in a 75-ml cell culture flask. Human Aortic Smooth Muscle Cells (HASMCs, passage 5) were suspended in smooth muscle cell growth media with supplement kit in a 75-ml cell culture flask. The cells were cultured inside a 37 °C and 5% CO_2_ incubator. Cell growth media were changed every other day until 90% confluence was attained. After the cell confluence was attained, cells were detached from the culture flask using TrypLE. The cell concentration was diluted to 20,000 cells/ml before cell seeding. All surfaces were sterilized with 70% ethanol incubation for 10 min. Then, samples were rinsed with DI water and PBS twice. Further, samples were exposed to UV light for 15 min. In a 48-well plate, sterilized surfaces were placed and 300 μl of diluted cells was pipetted into each well plates. The well plates were stored inside a 37 °C and 5% CO_2_ incubator for the duration of each individual study. Cell media was changed every other day.

### Cell toxicity

Cell toxicity of modified samples was quantified using lactate dehydrogenase (LDH) assay. After 24 h of incubation with HUVEC and SMC cells, 100 μl of incubated cell growth media was used to evaluate the LDH expression. Manufacturer provided protocol was followed, a mixture of 100 μl of assay reaction solution and 100 μl of incubated cell growth media was incubated in a 37 °C and 5% CO_2_ incubator for 30 min. After incubation, 50 μl of stop solution was added and the absorbance of the resulting solution at 490 nm using an UV spectrophotometer. Maximum LDH expression was evaluated by lysing solution (10% Triton X-100). Spontaneous LDH expression was evaluated by cells incubated in a blank well.

### Cell viability

Cell viability of modified samples was quantified using CellTiter-Blue Assay. After 1, 4, and 7 days of incubation with HUVEC and SMC, cell media was aspirated and 300 μl of 10% CellTiter-Blue was added to each well. The samples were further incubated in a 37 °C and 5% CO_2_ incubator for 7 h. Absorbance of the resulting solution 570 nm and 600 nm using an UV spectrophotometer. Manufacturer provided protocol was followed to calculate the viability.

### Cell adhesion and proliferation

Cell adhesion and proliferation of modified samples was visualized and quantified using fluorescence microscopy. After 1, 4, and 7 days of incubation with HUVEC and SMC, cell media were aspirated, and surfaces were gently rinsed with PBS thrice. The surface was fixed using a fixative solution (3.7% formaldehyde) for 15 min and the surfaces were gently rinsed with PBS thrice. The cells were permeabilized using membrane permeability solution (1% Triton X-100) for 3 min and surfaces were gently rinsed with PBS thrice. F-actin, actin filaments, and nucleus of the fixed cells were incubated in a staining solution (2% rhodamine-phalloidin) for 25 min and followed by a nucleus staining solution (3% 4′,6-diamidino-2-phenylindole) for 5 min. Surfaces were gently rinsed with PBS thrice and the surfaces were imaged using Zeiss fluorescence microscopy. Cells were quantified using ImageJ software. Specific protein markers of each cell type were quantified by measuring the percentage of area covered of stained proteins using ImageJ software.

### Cell morphology

Cell morphology of modified samples was visualized and quantified using fluorescence microscopy. After 1, 4, and 7 days of incubation with HUVEC and SMC, cell media was aspirated, and surfaces were gently rinsed with PBS thrice. The surface was fixed using a fixative solution (3% glutaraldehyde, 0.1 M sodium cacodylate, and 0.1 M sucrose) for 45 min followed by buffer solution (0.1 M sodium cacodylate and 0.1 M sucrose) for 10 min. The surfaces were consequently dried by incubating with 35%, 50%, 70%, and 100% ethanol for 10 min each. Surface adhered cell morphology was imaged using SEM.

### Cell differentiation

Cell differentiation of modified samples was visualized and quantified using fluorescence microscopy. After 5, 7, and 10 days of incubation with HUVEC and SMC, cell media were aspirated, and surfaces were gently rinsed with PBS thrice. The surface was fixed using a fixative solution (3.7% formaldehyde) for 15 min and the surfaces were gently rinsed with PBS thrice. The cells were permeabilized using membrane permeability solution (1% Triton X-100) for 3 min and surfaces were gently rinsed with PBS thrice. The samples were transferred to a new well and non-specific binding site and were blocked by incubating in 10% bovine serum albumin (BSA) for 30 min. VE-cadherin and vWF primary antibody for HUVEC and calponin and MYH for SMC (1:100 dilution) was added to the surfaces for 60 min at room temperature and the surfaces were rinsed three times with PBS. FITC secondary antibody (1:200 dilution) was then added to the surfaces for 45 min at room temperature and the surfaces were rinsed twice with PBS. F-actin, actin filaments, and nucleus of the fixed cells were incubated in a staining solution (2% rhodamine-phalloidin) for 25 min and followed by a nucleus staining solution (3% 4′,6-diamidino-2-phenylindole) for 5 min. Surfaces were gently rinsed with PBS thrice and the surfaces were imaged using Zeiss fluorescence microscopy. Cells were quantified using ImageJ software. Specific protein markers of each cell type were quantified by measuring the percentage of area covered of stained proteins using ImageJ software.

### Statistical analysis

Surface characterization was conducted on a minimum of three different surfaces from each group. Contact angle measurements, fluorescence microscopy, and SEM imaging were performed at three different locations on each sample, with a minimum of nine samples analyzed. Cytotoxicity, cell proliferation, adhesion, and differentiation assays were repeated at least two times, using a minimum of six different surfaces from each group. The quantitative data obtained were analyzed using a two-way analysis of variance (ANOVA) test. The results are denoted with asterisks (*) to indicate statistical significance, with a threshold of *p* < 0.05.

## Results and discussion

Patients diagnosed with heart diseases like atherosclerosis are implanted with metallic stents to restore flow of the blood. These stents are commonly made of metallic alloys such as stainless steel, and titanium, owning to their excellent mechanical properties and biocompatibility [23, 24]. However, upon implantation, the stent interacts with the inner layer of the blood vessel and damage the endothelial lining. This phenomenon is called endothelial denudation [34]. This causes the smooth muscle cells inside the blood vessels to interact with the stent surface, leading to de-differentiation and proliferation causing restenosis [35]. Researchers explored various surface modification techniques to improve surface hemocompatibility of these stent materials. DES have shown to prevent restenosis [36, 37]. However, after 5–6 months of implantation, the drug on the surface is exhausted and the surface interacts with the blood and leads to late thrombosis [38]. The key to prevent thrombosis is to promote endothelization. In this study, a simple hydrothermal treatment was explored to modify the surface. Modified surfaces were assessed for in vitro endothelization as this is found to prevent blood clotting and smooth muscle adhesion/proliferation as this is found to prevent restenosis.

### Surface characterization

**Fig. 1:**
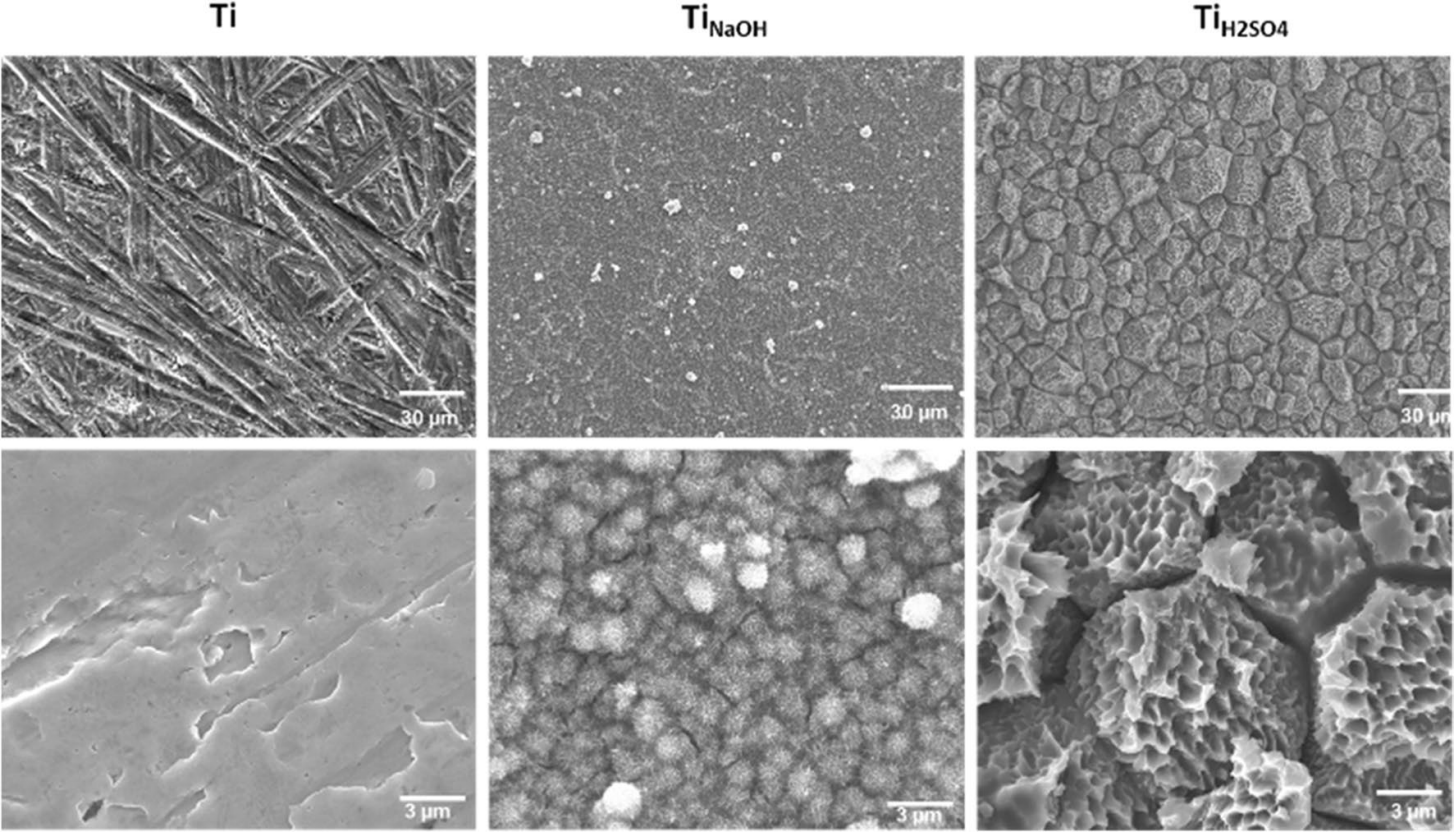
FE-SEM Images of polished and modified surfaces at two different magnifications

Surface morphology of the treated surfaces was visualized using a SEM. Polished Ti surfaces have planar surfaces and at high magnification polishing scratches were observed. After sodium hydroxide treatment, Ti_NaOH_ surface exhibited nanofibrous microstructure on the surface [24]. The formation of nanofibrous structure is attributed to the different etching rates between the grain and grain boundaries. The grain boundaries with weaker bonds were etched significantly higher than the grain surface. The etching inside the grain led to a fibrous structure as explained in previous manuscript [33]. In contrast, after sulfuric acid treatment, Ti_H2SO4_ surface showed multi-scalar features, and this was due to gradient etching. The etching rate was higher towards the grain boundaries and gradually decreased towards the center of the grain. The presence of highly disoriented atoms in the grain boundaries have led to the formation of 3D pyramid like micron structures (Fig. 1). In addition, unidirectional etching perpendicular to the surface has led to nano-pits [39]. The size and shape of the surface feature can modulate surface area available for cell adhesion. Surface was further characterized for chemistry (XPS) and crystal structure (XRD) this data has been published in author’s previous work [33].

**Fig. 2:**
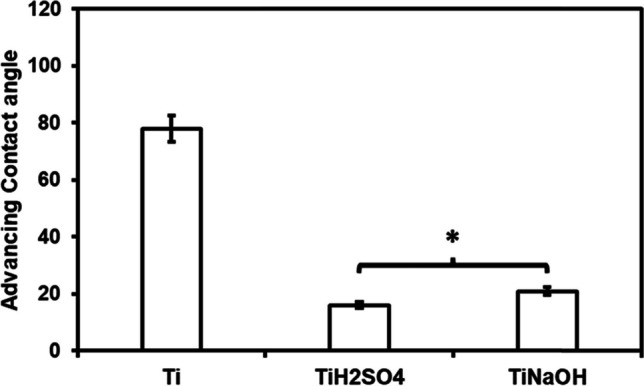
Advancing contact angle of different surfaces. Modified surfaces have significantly lower contact angle compared to control Ti. The error bar represents the standard deviation (*p < 0.05)

Surface wettability was characterized using a goniometer. Surface wettability plays a major role in protein adsorption and cell adhesion and hence was investigated. Advancing contact angle (θ_adv_) was measured using Milli-Q water. Advancing contact angle is the highest contact angle in the free energy range. Results indicated that all surfaces are hydrophilic (< 90°) with the trend for θadv: Ti < Ti_NaOH_ < Ti_H2SO4_ (Fig. 2). The advancing angle of Ti was significantly higher than Ti_NaOH_ and Ti_H2SO4_ due to the increased surface oxidation and surface area.

### Endothelial cell interaction with modified surfaces

The main functionality of endothelial cells inside a blood vessel is to regulate blood flow by regulating diameter of the blood vessel, inflammation, and coagulation. Size of the endothelial cells varies across the vascular tree, but they are in generally thin and slightly elongated, their dimensions are 50–70 μm long, 10–30 μm wide, and 0.1–10 μm thick. Endothelial cells can produce molecules like thrombomodulin and tissue factor pathway inhibitor (TFPI) that help to inhibit coagulation, while producing von Willebrand factor, which promotes platelet aggregation [40]. Endothelial glycocalyx is a layer of sugar molecules that surrounds the endothelial cells and lines the inner surface of the blood vessels. This layer is composed of a complex mixture of glycosaminoglycans, glycoproteins, and proteoglycans. The endothelial glycocalyx plays a crucial role in regulating blood flow by influencing the mechanical and chemical properties of the blood vessels [41].

**Fig. 3:**
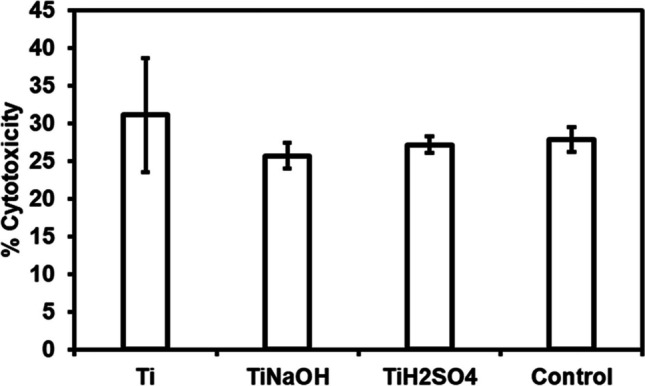
Cell cytotoxicity for HUVECs exposed to different surfaces after 24 h measured using the LDH assay. The error bar represents the standard deviation

Hydrothermal treatment modifies surface properties such as morphology, chemistry, and wettability. Hence, it is vital to evaluate surface induced toxicity. HUVECs when induced to toxic environment, stop proliferating and slowly die. During this process, cells will lose membrane integrity and release various enzymes into the medium. LDH is a dominant enzyme found when cells die due to apoptosis and necrosis. Apoptosis is due to natural cell death as part of an organ growth. Necrosis is due to environment induced cell death [42]. Modified surfaces were evaluated for cytocompatibility after incubating with HUVECs for 24 h using commercially available LDH assay. Results indicated that hydrothermal treatment did not induce toxicity as there was no significant difference between the toxicity levels (Fig. 3) with cells incubated with Ti, control (polystyrene well), Ti_NaOH_ and Ti_H2SO4_.

**Fig. 4:**
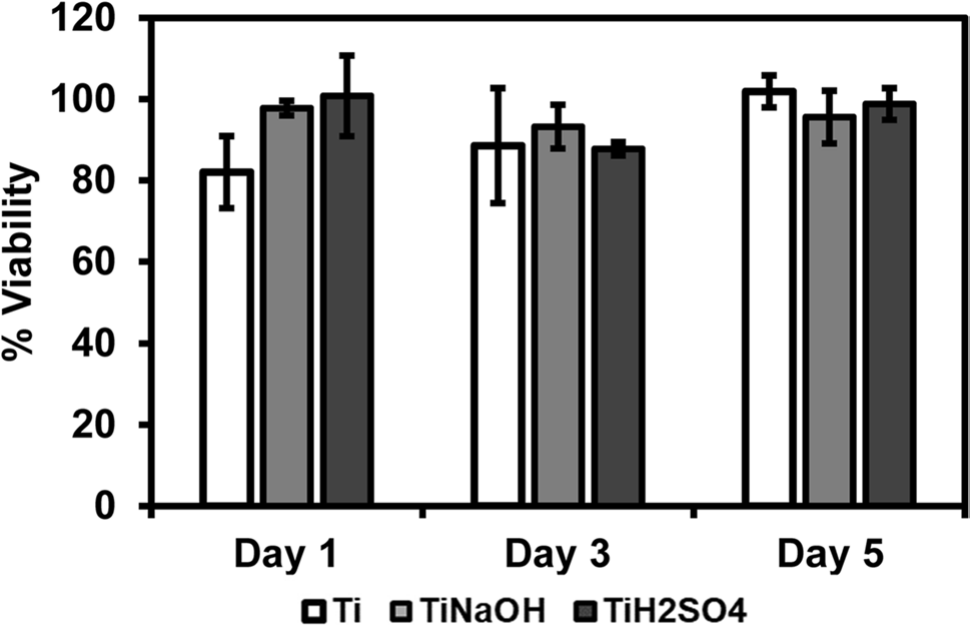
Cell viability for HUVECs exposed to different surfaces measured after days 1, 3, and 5 of incubation using the cell viability assay. The error bar represents the standard deviation

Surfaces were evaluated for cell viability after incubating with HUVECs for 1, 3, and 5 days using commercially available CellTiter-Blue assay. Hydrothermal treatment modifies surface properties such as morphology, chemistry, and wettability and this can influence cell interactions. Hence, it is vital to evaluate modified surface for cell adhesion and proliferation. Metabolically active cells will interact with resazurin present in the reagent and reduces to resorufin. Higher resorufin in the resulting solution indicates more viable cells [43]. Results indicate that after 1 day of incubation, no significant difference was seen between Ti, Ti_NaOH_, and Ti_H2SO4_. Similarly, no significant difference was seen after 3 and 5 days of incubation. However, Ti_H2SO4_ had a significant increase cell proliferation after 3 and 5 days of incubation (Fig. 4). Ti_NaOH_ showed the fastest growth between 1 and 3 days of incubation.

**Fig. 5:**
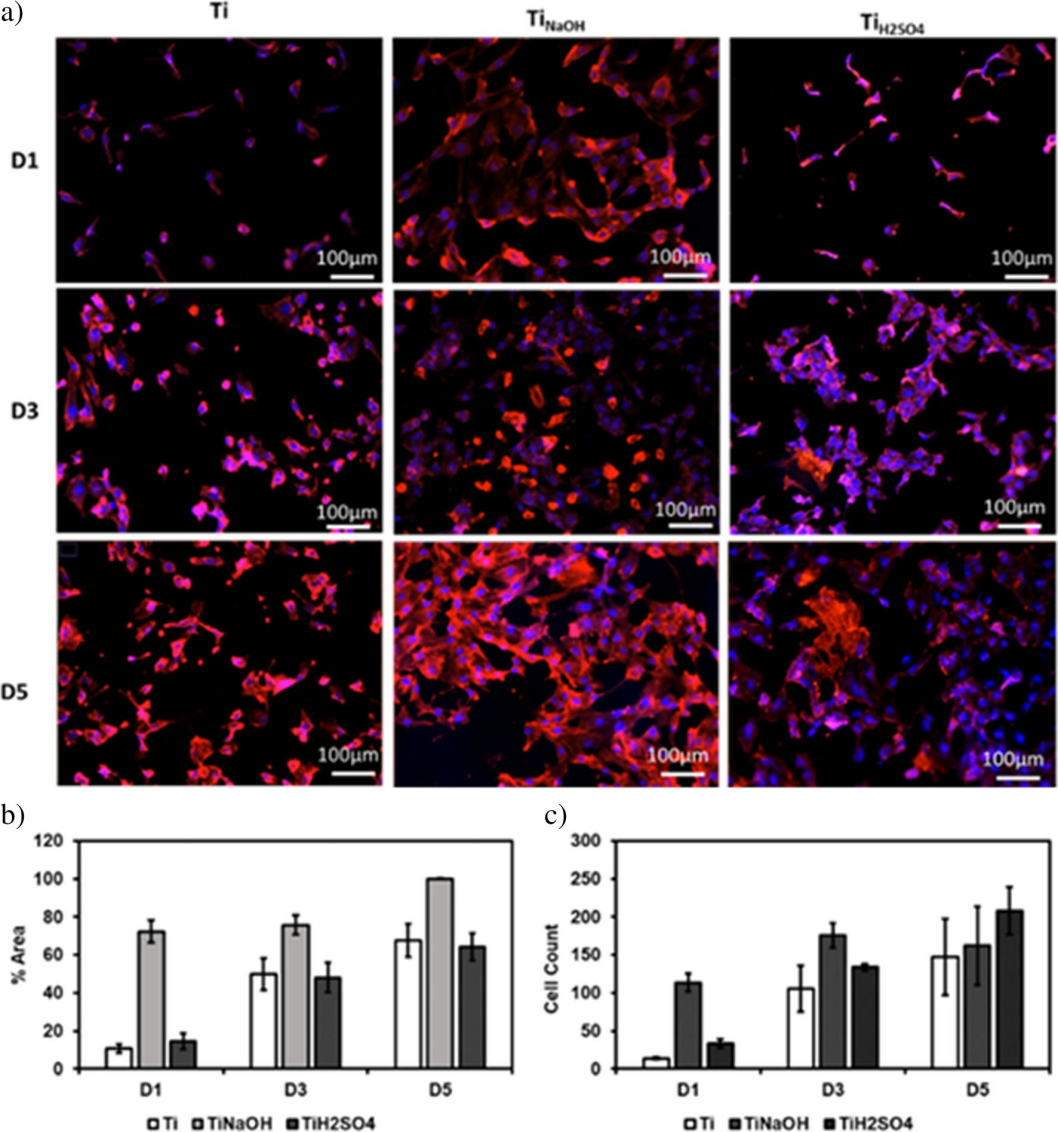
**a** Fluorescence microscope images HUVECs stained with DAPI and rhodamine–phalloidin on different surfaces after days 1, 3, and 5 of incubation. **b** Percentage area coverage of cells on different surfaces after 1, 3, and 5 of incubation. **c** Number of cells adhered to the surface stained with DAPI calculated using ImageJ. The error bar represents the standard deviation (*p < 0.05)

Cell adhesion and proliferation of the modified surfaces after incubating with HUVECs for 1, 3, and 5 days were evaluated using a fluorescence microscope. Hydrothermal treatment modifies surface properties such as morphology, chemistry, and wettability, and this in turn modifies the cell adhesion and proliferation [44]. Hence, it is vital to evaluate if modified surfaces promote cell adhesion and proliferation as they can prevent early thrombosis and restenosis. Results indicate that after 1 day of incubation, Ti_NaOH_ showed significantly higher cell adhesion compared to Ti_H2SO4_ and Ti surface. Similar trend was observed after 3 and 5 days of incubation (Fig. 5a). However, all surfaces showed significant increase in cell coverage between the different time points (Fig. 5b). Cell count studies showed that Ti_NaOH_ had significantly higher cell on day 1 and 3 compared to Ti_H2SO4_ and Ti. However, cell count studies after 5 days of incubation showed that Ti_H2SO4_ had higher cells compared to Ti_NaOH_ and Ti (Fig. 5c). This is mainly due to the interaction of cells with different surface morphology, endothelial cells are smaller in size and cuboidal (roughly square under a microscope). Hence, the hydrophilic Ti_H2SO4_ surface with the micro-nano-features promotes cell adhesion and proliferation growth although its micro-nano-surface prevents cell spreading. Thus, after 5 days of incubation, Ti_H2SO4_ has a higher cell count with lower cell coverage compared to Ti_NaOH_.

**Fig. 6:**
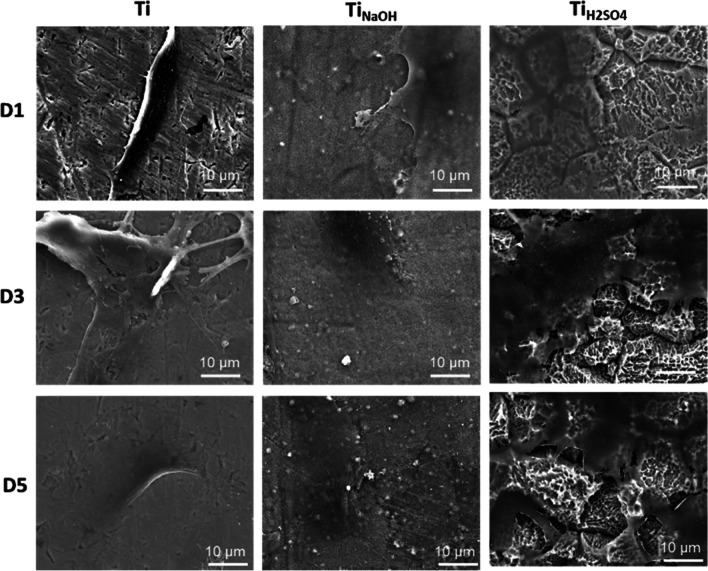
SEM images of adhered cells on different surfaces after day 1, 3, and 5 of incubation. Images were taken at 1000× magnifications

Surfaces were evaluated for cell morphology after incubating with HUVECs for 1, 3, and 5 days using a SEM. Hydrothermal treatment modifies the surface properties such as morphology, chemistry, and wettability. Hence, it is vital to evaluate if modified surfaces influence cell morphology. Results indicated that Ti surface enables the cells to spread and Ti_NaOH_ surface with planar fibrous nano-structures promote higher cell spreading (Fig. 6). However, the Ti_H2SO4_ surface with micron scale pyramid features prevents cell spreading significantly and this corroborates with the cell adhesion and proliferation results.

**Fig. 7:**
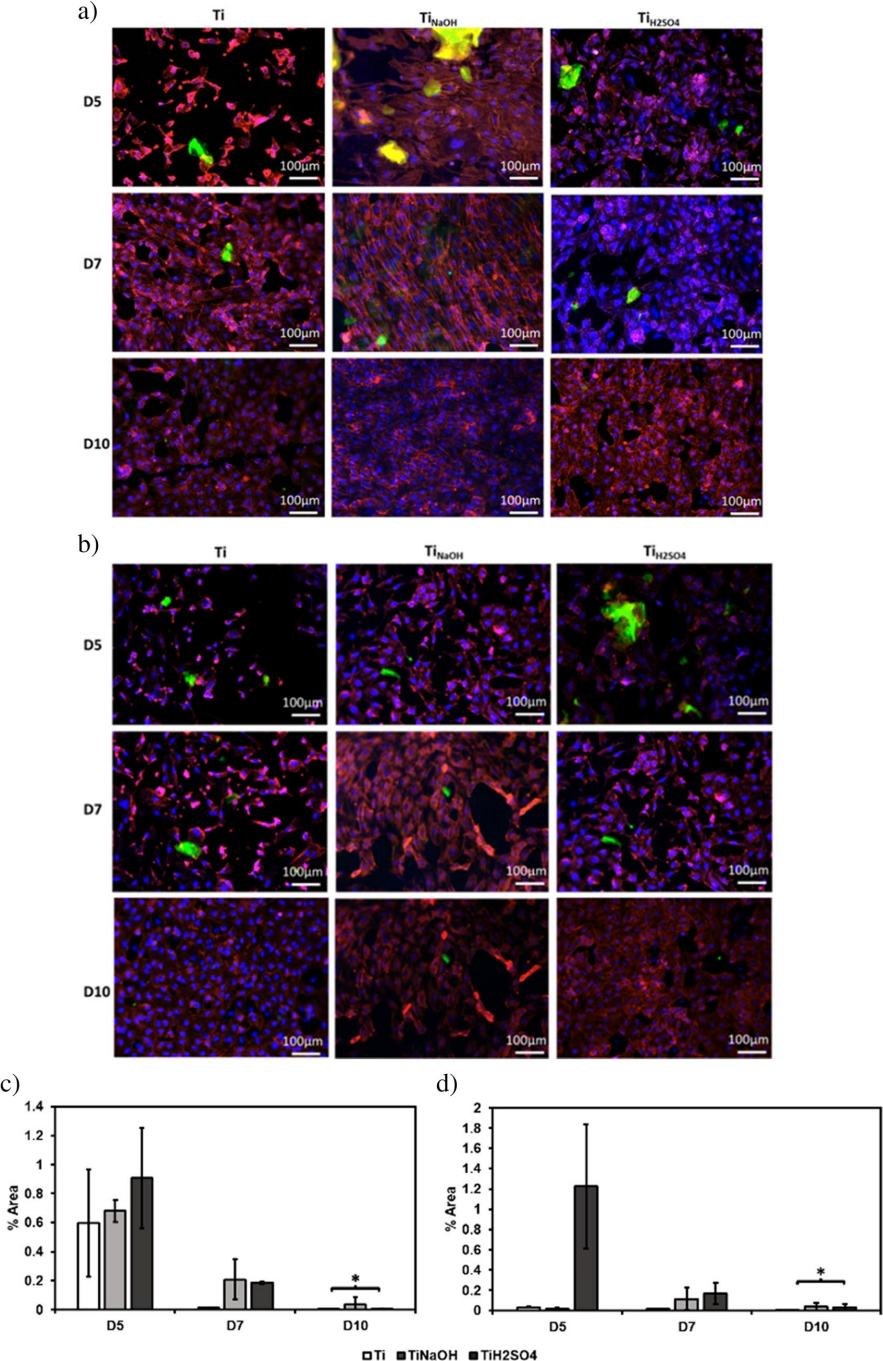
Fluorescence microscope images of HUVECs along with **a** VE-cadherin and **b** vWF after days 5, 7, and 10 of incubation with DAPI and rhodamine–phalloidin on different surfaces. **c** Percentage area coverage of VE-cadherin after days 5, 7, and 10 of incubation. d Percentage area coverage of vWF after day 5,7 and 10 of incubation (*p < 0.05)

Surfaces were evaluated for protein expression after incubating with HUVECs for 5, 7, and 10 days using an immunofluorescence staining assay. Hydrothermal treatment modifies surface properties such as morphology, chemistry, and wettability. Hence, it is critical to evaluate if modified surfaces influence protein expression. Although implant surface has to adhere and assist cell proliferation, it is crucial for the developed surface to support protein expression to maintain a healthy blood vessel. HUVECs during healthy proliferation produce certain proteins such as vascular endothelial cadherin (VE-cadherin), and von Willebrand factor (vWF). Hence, the presence of such protein markers confirms endothelialization with the modified surface. After 7 days of incubation, there was a significant increase in endothelization on both Ti_NaOH_ and Ti_H2SO4_ compared to Ti. After 10 days of incubation, the cells have changed to cuboidal shapes on all surfaces, cuboidal shape of endothelial cells allows for a high surface-to-volume ratio, which is important for the many physiological functions (Fig. 7a, b).

VE-cadherin is a type of cadherin protein that is expressed after endothelization. It is important for maintaining the integrity of the blood vessels by holding the endothelial cells together and preventing the leakage of blood from the vessels. It also plays a role in the formation and stability of the blood vessels during development and tissue repair. VE-cadherin can modulate the activity of intracellular signaling pathways that control the smooth muscle cells, altering the diameter of the blood vessels. Results indicated Ti_NaOH_ and Ti_H2SO4_ initiated protein expression (Fig. 7c).

vWF is a glycoprotein that plays a crucial role in blood clotting. vWF is produced during endothelization and stored in the endothelial cells and platelets and is released into the bloodstream in response to injury or inflammation. During a blood vessel injury, the exposed subendothelial collagen activates platelets, which in turn release vWF from their storage granules. vWF acts as a bridge between platelets and the collagen, helping platelets to stick to the site of injury and aggregate to form a platelet plug. vWF also acts as a carrier protein for clotting factor VIII, which is essential for the formation of a stable clot. Results indicated Ti_H2SO4_ initiated faster protein expression (Fig. 7d). Thus, the studies show that all surfaces are able to differentiate the HUVECs completely.

### Smooth muscle cell interaction with modified surfaces

The main functionality of SMCs in a blood vessel is to repair vasculature after injury. Size of SMCs varies across the vascular tree, but they are in generally bigger than endothelial cells with a large nucleus in the center, their average dimensions are around 200 μm long and 5 μm wide. However, these properties can act disadvantageous by reacting abnormally to post-injuries and lead to restenosis. Hence, surfaces which promote SMCs adhesion, proliferation, and de-differentiation can alleviate restenosis. Thus, it is crucial to develop surfaces that selectively promote EC adhesion while maintaining differentiated states of SMCs for implant longevity.

**Fig. 8:**
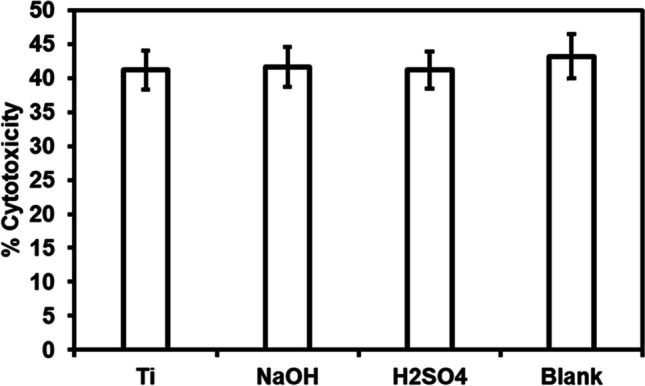
Cell cytotoxicity for SMCs exposed to different surfaces after 24 h measured using the LDH assay. The error bar represents the standard deviation

Surfaces were evaluated for cytocompatibility after incubating with SMCs for 24 h using commercially available LDH assay. Results indicated that hydrothermal treatment did not induce toxicity as there was no significant difference between the toxicity levels with SMCs (Fig. 8) incubated with Ti, control (polystyrene well), Ti_NaOH_, and Ti_H2SO4_.

**Fig. 9:**
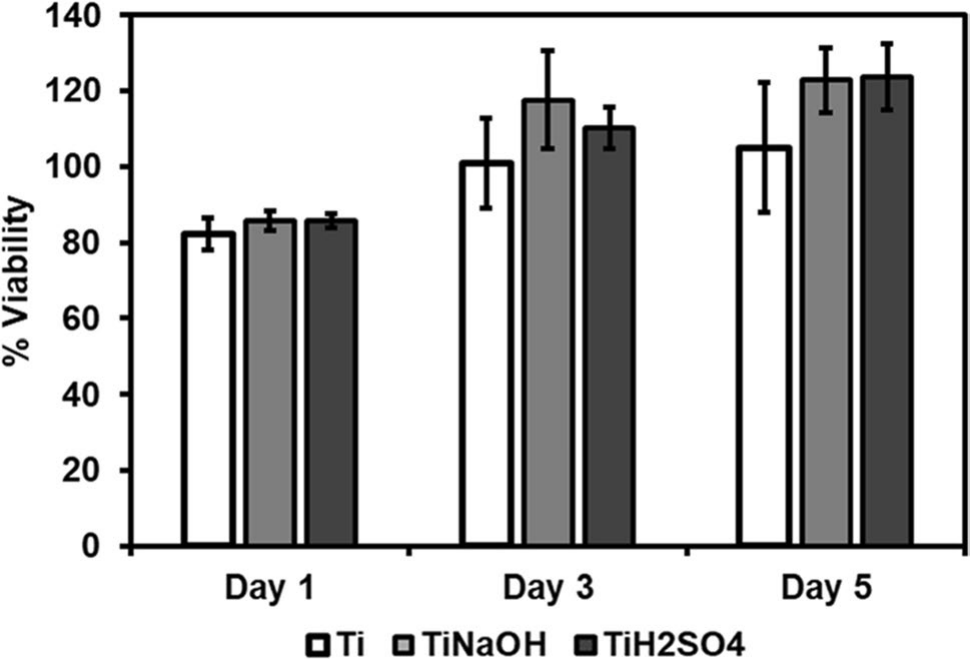
Cell viability for SMCs exposed to different surfaces measured after days 1, 3, and 5 using the cell viability assay. The error bar represents the standard deviation

Surfaces were evaluated for cell viability after incubating with HUVECs for 1, 3, and 5 days using commercially available CellTiter-Blue assay. Results indicate that after 1, 3, and 5 days of incubation, no significant difference was seen between Ti, Ti_NaOH_, and Ti_H2SO4_. Similarly, no significant difference was observed between the different points (Fig. 9). This implies that SMC growth rate on different surfaces was in par with cell growth with the control well (polystyrene).

**Fig. 10:**
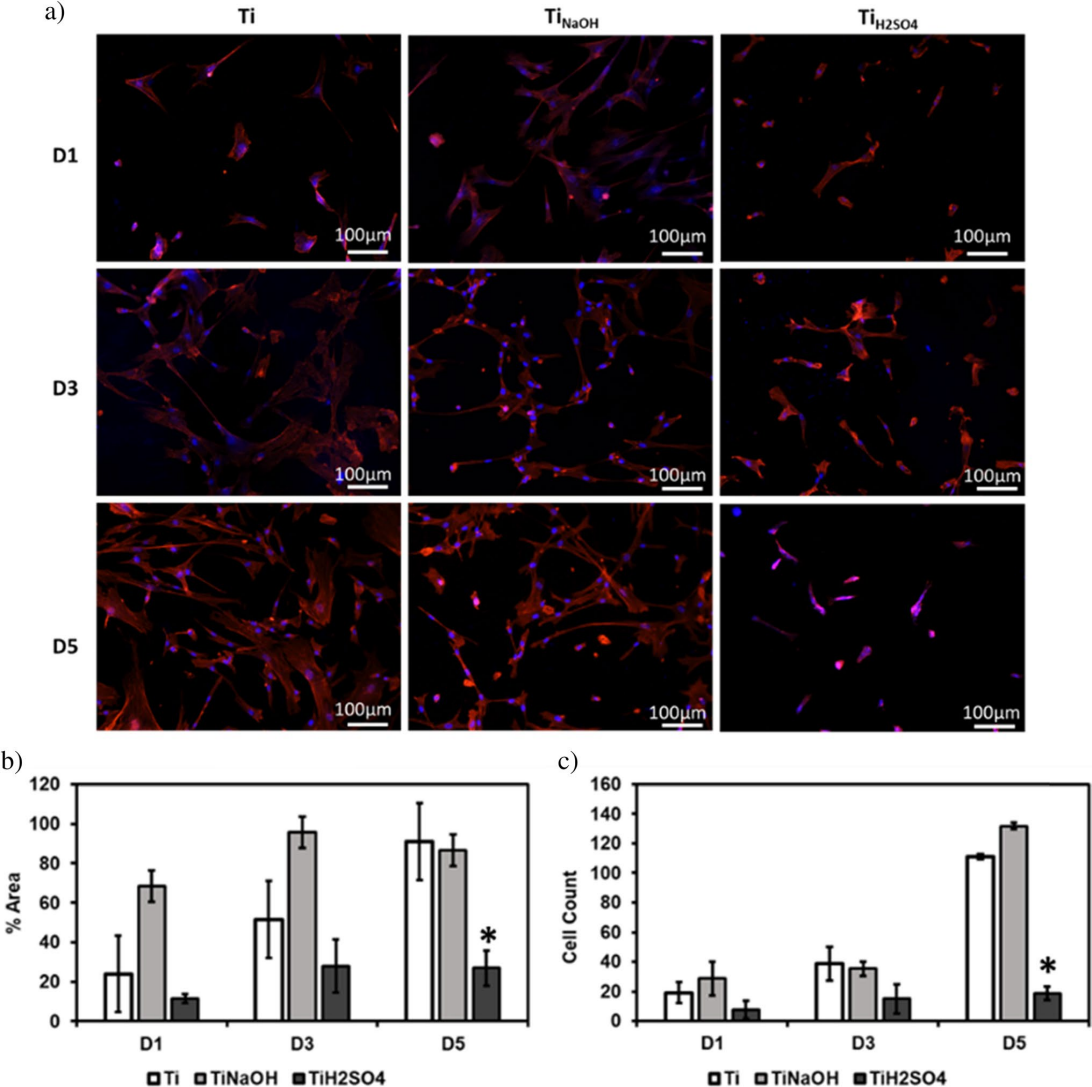
**a** Fluorescence microscope images SMCs stained with DAPI and rhodamine–phalloidin on different surfaces after day 1, 3, and 5 of incubation. **b** Percentage area coverage of cells on different surfaces after 1, 3, and 5 of incubation. **c** Number of cells adhered to the surface stained with DAPI calculated using ImageJ. The error bar represents the standard deviation (*p < 0.05)

Surfaces were evaluated for cell adhesion and proliferation after incubating with SMCs for 1, 3, and 5 days using a fluorescence microscope. Results indicate that after 1 day of incubation, Ti_NaOH_ showed significantly higher cell adhesion compared to Ti_H2SO4_ and Ti surface. Similar trend was observed after 3 and 5 days of incubation (Fig. 10a). Similarly, the Ti surface showed a significant increase in cell coverage between the different time points (Fig. 10b). However, Ti_H2SO4_ showed no significant increase in cell count between different time points. Cell count studies showed that cells had significantly higher cells on day 1, 3, and 5 compared to Ti_H2SO4_ and Ti. Ti_H2SO4_ surface had the lowest cells compared to Ti_NaOH_ and Ti (Fig. 10c). This is mainly due to the interaction of cells with different surface morphology, Ti_H2SO4_ with the micro-nano-surface features prevents bigger SMCs spreading and proliferation. This is because SMCs are larger in size, and they need larger surface area to adhere and spread to proliferate and Ti_H2SO4_ micron scale polygon features prohibit spreading. Thus, Ti_H2SO4_ has the lowest SMCs adhesion and proliferation compared to Ti_NaOH_ and Ti.

**Fig. 11:**
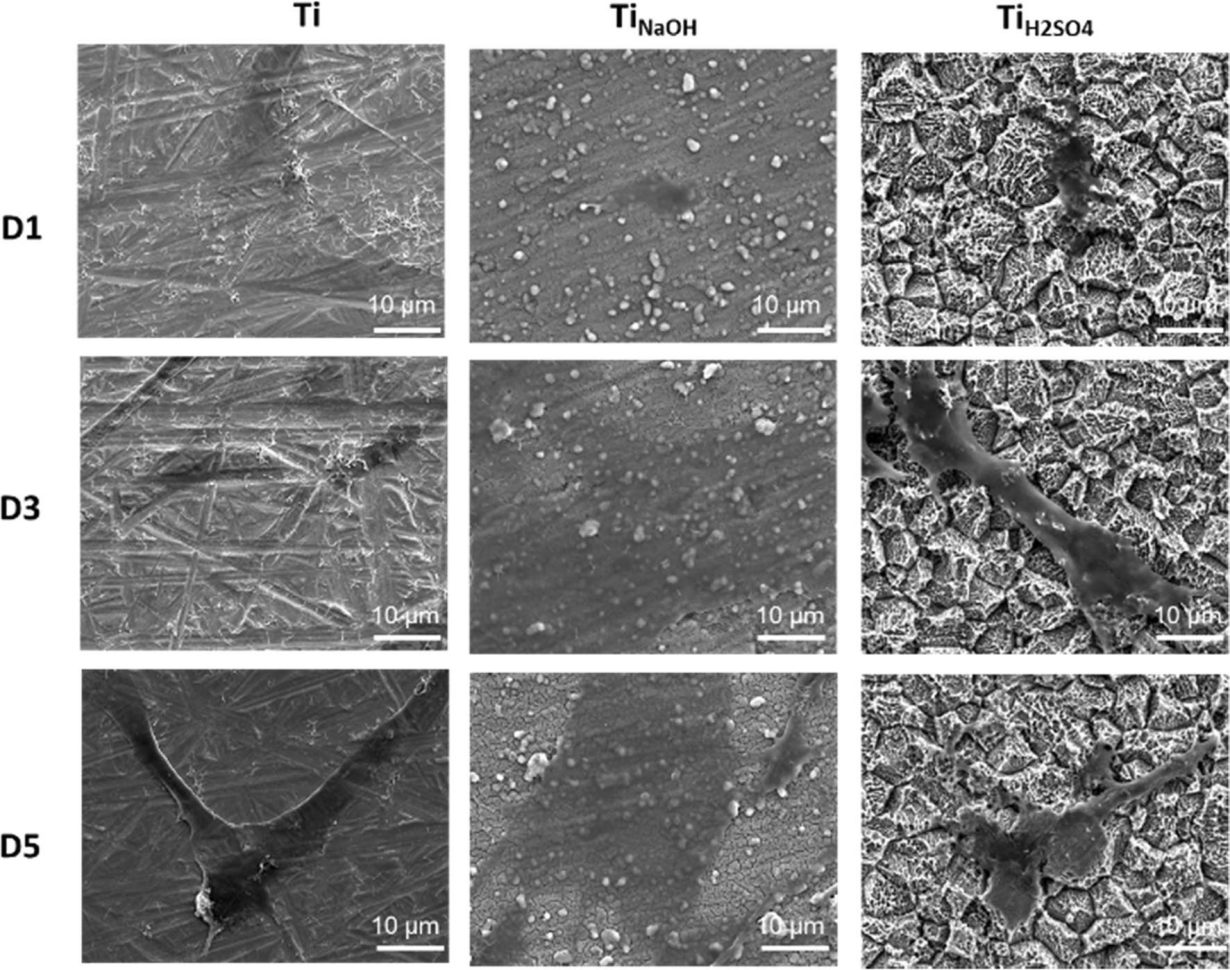
SEM images of adhered cells on different surfaces after days 1, 3, and 5 of incubation. Images were taken at 1000× magnifications

Surfaces were evaluated for cell morphology after incubating with SMCs for 1, 3, and 5 days using a SEM. Images were taken at high magnification to understand the cell morphology. Results indicated that Ti surface enables the cells to spread and Ti_NaOH_ surface with planar fibrous nano-structures promotes higher cell spreading. However, the Ti_H2SO4_ surface with micron scale pyramid features restricted cell spreading significantly and this corroborates with the cell adhesion and proliferation results (Fig. 11). Thus, Ti_H2SO4_ with lower the cell adhesion/spreading will have lower chances for restenosis compared to Ti and Ti_NaOH_.

**Fig. 12:**
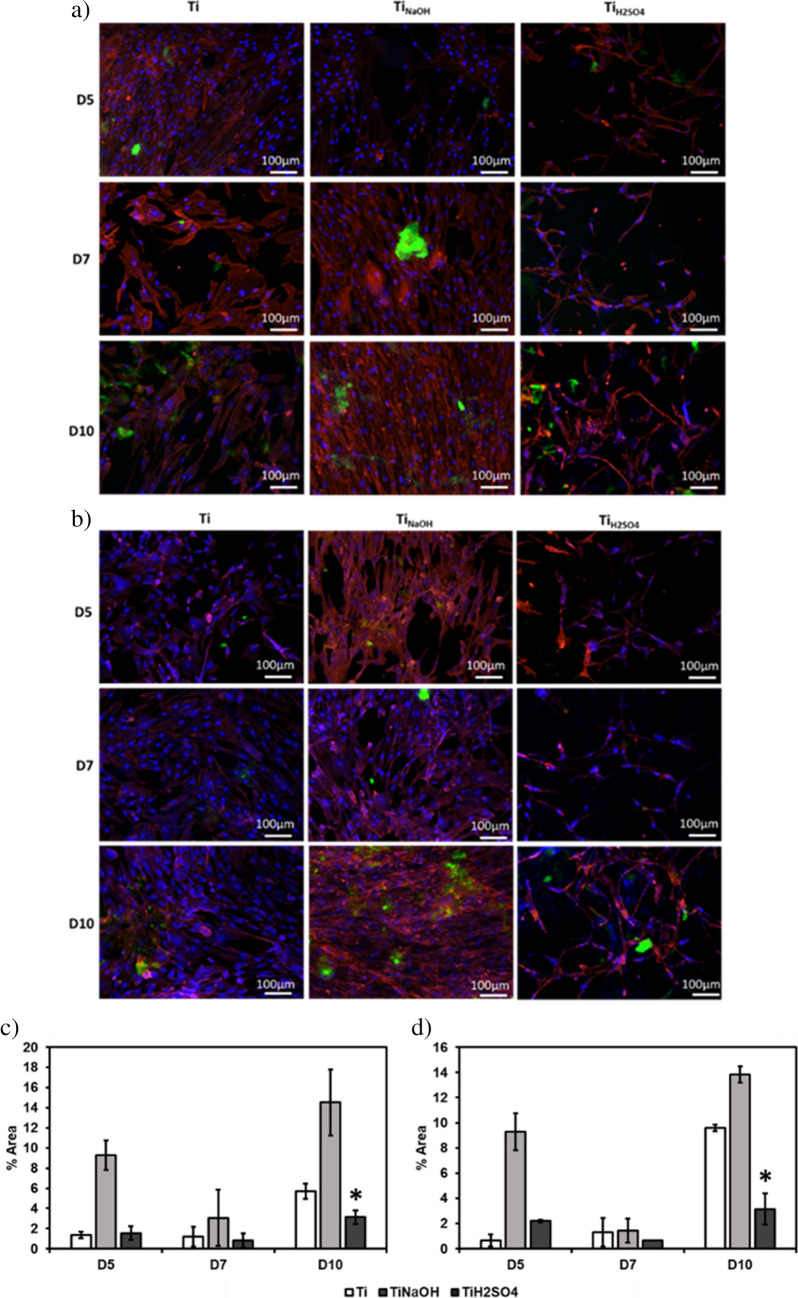
Fluorescence microscope images of SMCs along with **a** calponin and **b** MYH after days 5, 7, and 10 of incubation stained with FITC, DAPI, and rhodamine–phalloidin on different surfaces. **c** Percentage area coverage of calponin after days 1, 3, and 5 of incubation. d Percentage area coverage of MYH after days 1, 3, and 5 of incubation. The error bar represents the standard deviation (*p < 0.05)

Surfaces were evaluated for cell differentiation after incubating with SMCs for 5, 7, and 10 days using an immunofluorescence staining assay. Recent genetic lineage tracing studies have shown that SMC phenotypic switching directly promotes atherosclerosis. Results indicated Ti_NaOH_ initiated faster cell adhesion and spreading compared to Ti_H2SO4_ and Ti throughout the entirety of the study (Fig. 12a, b).

Calponin is a protein that is found abundant in smooth muscle cells, it regulates cell contraction and it is considered a reliable marker for SMC differentiated state. Result indicated that Ti_NaOH_ surface had highest calponin protein expression at all time points and Ti_H2SO4_ surface had significantly lower calponin expression after 10 days of incubation.

Myosin heavy chain (MYH) is a cytoplasmic structural protein present in SMCs; it influences the mechanical properties of the cells. This is expressed during SMC development and differentiation. Result indicated a similar trend as compared to calponin. The presence of these protein markers indicates that the SMC are not de-differentiating. Hence, the modified surfaces are not promoting SMC de-differentiation and prevents restenosis. Ti_H2SO4_ had the lowest cell adhesion and thus lower expression of both calponin and MYH at different timepoints compared to Ti_NaOH_ and Ti (Fig. 12c, d).

## Conclusion

The single layer of endothelial cells lining the blood vessels plays a major role in maintaining vascular hemostasis. After stenting procedure, significant injuries are done to the endothelial cell layer and these injuries lead to inflammation and development of neo-intimal hyperplasia. Restenosis after stent implantation has been primarily due to smooth muscle cell proliferation. Hence, it is crucial to develop an implant surface which prevents smooth cell adhesion and proliferation. Previous work by the author has shown improved antithrombogenic properties, with reduced platelet adhesion and activation on sulfuric acid treated surface compared to smooth titanium surface, which indicates they are promising candidates for cardiovascular implant applications. Titanium surfaces are hydrothermally treated with sodium hydroxide and sulfuric acid separately to develop two different surface morphologies. Titanium treated with sodium hydroxide solution led to a planar fibrous surface with etched grain boundaries. Titanium treated with sodium hydroxide solution led to a 3D-like microscale pyramidical structures with nanoscale pits. Surface wettability studies showed that both hydrothermally treated surfaces were significantly hydrophilic compared to control titanium surface. Surfaces were evaluated for cytocompatibility, cell viability, adhesion, proliferation, and differentiation with both endothelial cells and smooth muscle cells. Modified surfaces did not induce any toxicity after 24 h of HUVECs and SMCs incubation. Cell adhesion and proliferation studies showed that sulfuric acid treatment prevented smooth muscle cells proliferation. However, this surface promoted endothelial cell adhesion and proliferation as the surface features were bigger than endothelial cells and therefore did not influence smaller endothelial cells. Differentiation studies showed that the sulfuric acid treated surface had higher endothelial cell differentiation on the surface with phenotypic changes in cell morphology. Hence, these surfaces have potential of being used for cardiovascular implants.
